# Intimate Partner Violence and Pregnancy Termination in Armenia: Evidence from Nationally-Representative Survey Data

**DOI:** 10.3390/ejihpe11020022

**Published:** 2021-03-28

**Authors:** Nandeeta Samad, Pranta Das, Bright Opoku Ahinkorah, Abdul-Aziz Seidu, James Boadu Frimpong, Joshua Okyere, John Elvis Hagan, Mohammad Hayatun Nabi, Mohammad Delwer Hossain Hawlader

**Affiliations:** 1 Department of Public Health, School of Health and Life Sciences, North South University, Dhaka 1229, Bangladesh; nandeeta.samad@northsouth.edu (N.S.); mohammad.nabi@northsouth.edu (M.H.N.); mohammad.hawlader@northsouth.edu (M.D.H.H.); 2 Department of Statistics, University of Dhaka, Dhaka 1000, Bangladesh; pranta.du.stat@gmail.com; 3 Faculty of Health, School of Public Health, University of Technology Sydney, Sydney, NSW 2007, Australia; bright.o.ahinkorah@student.uts.edu.au; 4 Department of Population and Health, Faculty of Social Sciences, College of Humanities and Legal Studies, University of Cape Coast, Cape Coast, PMB TF0494, Ghana; abdul-aziz.seidu@stu.ucc.edu.gh (A.-A.S.); joshua.okyere@stu.ucc.edu.gh (J.O.); 5 College of Public Health, Medical and Veterinary Services, James Cook University, Townsville, QLD 4811, Australia; 6 Department of Health, Physical Education, and Recreation, University of Cape Coast, Cape Coast, PMB TF0494, Ghana; james.frimpong@stu.ucc.edu.gh; 7 Neurocognition and Action-Biomechanics-Research Group, Faculty of Psychology and Sports Science, Bielefeld University, Postfach 10 01 31, 33501 Bielefeld, Germany

**Keywords:** Armenia, emotional violence, intimate partner violence, physical violence, pregnancy termination, sexual violence

## Abstract

Intimate partner violence has been associated with numerous consequences for women, including pregnancy termination. This study examined the association between predictive capacity of intimate partner violence and pregnancy termination among women in Armenia. The study analyzed the 2015–16 Armenia Demographic and Health Survey (ADHS) data on women aged 15–49 (Mean: 31.49; Standard Deviation, SD: 9.51). Marital control exercised by husbands, ever experienced physical violence, sexual violence, and emotional violence by husbands were the four indicators of intimate partner violence used in this study. To assess the association between intimate partner violence and pregnancy termination, a binary logistic regression model was fitted. After controlling for confounders, we found that women whose husbands exercised marital control were 26% more likely to experience pregnancy termination, compared to women whose husbands did not exercise marital control (adjusted odds ratio (aOR): 1.26, 95% Confidence interval (CI): 1.03–1.53). Women who ever experienced sexual violence were about 10 times likely to experience pregnancy termination than women who did not experience sexual violence (aOR: 9.76, 95% CI: 1.91–49.96). Both ever experienced physical violence and emotional violence did not have any significant associations with pregnancy termination. Forms of intimate partner violence are associated with pregnancy termination. The findings of this study provide evidence for government and policymakers to formulate, modify, and implement policies and program that target both men and women regarding the prevailing intimate partner violence and its consequences. Strengthening the policy implementation will ensure that women are empowered to make decisions about their reproductive health. Making husbands and their family members aware of the basics and consequences of intimate partner violence and focusing on child cognitive development which can be hampered due to the prevalence violence in families are recommended.

## 1. Introduction

Pregnancy termination has been identified as one of the major public health issues worldwide [1]. According to the World Health Organization (WHO), nearly 56 million abortion cases are recorded globally, with legal abortion contributing to about 34 million of these figures annually [1]. Unlike other low- and middle-income countries, in most European countries, including Armenia, pregnancy termination is legalized. Notwithstanding the legal abortion services in Armenia for over five decades, the quality of these abortion services is faced with numerous challenges [2]. For example, it was reported that abortion accounts for about 12.5% of maternal deaths in Armenia [2,3]. In this light, medical abortion guidelines and medications were introduced to reduce further the rate of maternal deaths associated with abortion, yet little impact was made [2]. Moreover, given the existing law regarding pregnancy termination, many women go in for abortions when they are faced with pregnancy complications [4].

Intimate partner violence (IPV) or women abuse involves inflicting or threatening to inflict pain on a female partner whether psychologically, physically, or sexually [5,6]. In recent times, intimate partner violence issues have gained significant feet in national development across the globe [7]. However, without doubt, the situation lingers on, even in European countries, like Armenia [5,7]. For instance, it was reported that one -third of women are more likely to experience physical or sexual violence in Armenia [5]. Moreover, the Armenian security service records 784 violence cases and receives over 2000 calls regarding women abuse yearly [8], which indicates that violence against women is endemic in Armenia.

Even though there exist a number of studies that have examined the prevalence of intimate partner violence and pregnancy termination from diverse contextual backgrounds [9,10,11,12,13,14,15,16], our comprehensive search revealed that no study had examined the association between intimate partner violence and pregnancy termination among Armenian women. All the facts mentioned above present a gap in the literature that ought to be addressed.

Therefore, the current study examined the predictive capacity of intimate partner violence and pregnancy termination among women in Armenia, using data from the 2015–16 Armenia Demographic and Health Survey (ADHS). Findings from this study will suggest ways of reducing pregnancy termination, which, in the long-term, will contribute to a further reduction in global maternal mortality and enhance gender equality worldwide as targeted in the United Nation’s Sustainable Development Goals (SDGs).

The socio-cultural model on IPV, which combines elements of family systems theory, social learning theory, social structures, and cultural factors to explain IPV, forms the theoretical basis for this study. Straus and Hotaling [17] first developed it. This model places IPV in the context of a high level of violence in our culture, the sexist organization of our society and family systems, and cultural norms legitimizing violence against family members. According to this model, family interactions inherently contribute to violent behaviors, particularly as a result of the manifestation of these societal influences at the level of family structure, norms of parental behavior and child-rearing, and individual interactions [17].

## 2. Materials and Methods

### 2.1. Data Source and Study Design

The study analyzed the 2015–16 ADHS data, which was published from 8 December 2015 to 5 April 2016. The 2015–16 ADHS, implemented by the National Statistical Service (NSS) and Ministry of Health (MOH) of the Republic of Armenia, used the Armenia Population and Housing Census as the sampling frame. The sampling frame consisted of the enumeration areas (EAs) covering the whole country. The 2015–16 ADHS sample was selected in two stages. In the first stage, from a list of EAs, 313 clusters comprising 192 in urban areas and 121 in rural areas were selected. In the second stage, from each selected cluster, a complete listing of households was carried out. Then, households were systematically selected for the interview. All women aged 15–49 (Mean: 31.49; Standard Deviation, SD: 9.51), regardless of whether a permanent resident or not, were eligible for the interview. In addition, one sub-sample of eligible women was randomly selected from each household to be interviewed regarding domestic violence.

### 2.2. Study Variables

#### 2.2.1. Outcome Variable

Whether the individual ever had pregnancy that terminated in the form of miscarriage, abortion, or stillbirth was the outcome variable of the study. The variable was coded as ‘Yes’ if the individual ever had a pregnancy terminated; otherwise, it was ‘No’.

#### 2.2.2. Exposure Variables 

Marital control exercised by husband, ever experienced physical violence, sexual violence, and emotional violence by husband were the four exposures for this study. The exposure, marital control exercised by husband, was coded from five indicators: (a) husband jealous if talking with other men, (b) husband accuses her of unfaithfulness, (c) husband does not permit her to meet her girl-friends, (d) husband tries to limit her contact with family, and (e) husband insists on knowing where she is. This exposure was coded as ‘Yes’ if the husband exercised any of the five indicators; otherwise, it was ‘No’. The exposure, ever experienced physical violence by husband, was coded as ‘Yes’ if husband ever pushed, shook or threw something, or husband ever slapped, or husband ever punched with fist or something harmful, or husband ever kicked or dragged, or husband ever tried to strangle or burn, or husband ever threatened with knife/gun or other weapon, or husband ever attacked with knife/gun or other weapon; otherwise, it was coded as ‘No’. Experience of sexual violence by husband was coded as ‘Yes’ if the individual ever experienced any of the following any situations- husband ever physically forced sex when not wanted, husband ever forced other sexual acts when not wanted; otherwise, it was coded as ‘No’. Experience of emotional violence by husband was coded as ‘Yes’ if the husband ever humiliated her, or husband ever threatened her with harm, or husband ever insulted her or made her feel bad; otherwise, it was coded as ‘No’.

#### 2.2.3. Confounding Variables 

Age group (15–24, 25–34, ≥35), residence (urban, rural), education level (primary or less, secondary, higher), wealth index (poorest, poorer, middle, richer, richest), contraception use (yes, no), employment status (employed, unemployed), reads newspaper or magazine (yes, no), and number of living children (none, one, two, three or more) were the confounding variables selected for this study from previous studies [10,15,18]. 

### 2.3. Statistical Analyses

The data of 2845 women who were married and usual residents of the household were analyzed after removing all the missing and “don’t know” responses. Design adjusted chi-square test was conducted to see the relationship between outcome variable and all the covariates independently. Binary logistic regression models were fitted to individually examine the relationship of each exposure variable with the outcome variable in the presence of all the confounders. Multicollinearity of the model was assessed by calculating the variance inflation factor (VIF). Goodness of fit of each model was assessed by conducting Hosmer-Lemeshow goodness of fit test and by calculating Nagelkerke’s pseudo R-square. All the analysis was done after incorporating the survey weight and the complex survey design. All the *p*-values less than 0.05 were considered significant. Software R version 4.0.3 was used to conduct all the analysis.

### 2.4. Ethical Approval and Consent to Participate

The survey was conducted by the National Statistical Service and the Ministry of Health of Armenia, along with the technical support by Inner City Fund (ICF) International and funding from United States Agency for International Development (USAID), the United Nations Population Fund (UNFPA), the Joint United Nations Program on HIV/AIDS (UNAIDS), and the United Nations Children’s Fund (UNICEF) with approval from Institutional Review Board (IRB). The 2015–2016 ADHS maintained written consent from the household heads during the survey. Informed consent was always taken from each respondent before enrolling them. Approval from ICF was obtained in November 2020 to access the data set from the Demographic and Health Survey (DHS) online archive.

## 3. Results

### 3.1. Characteristics of the Respondents

In the sample, most of the respondents were in age group > 35, and most of them were from urban areas. Regarding education level of the respondents, most of them had higher level of education and a least number of the respondents had primary or less level of education. Regarding wealth status, most of the respondents belonged to the richest wealth status. More comprehensive results regarding the characteristics of the respondents are presented in Table 1.

### 3.2. Distribution of the Prevalence of Pregnancy Termination

Pregnancy termination was more prevalent in the age group ≥35, in rural people, in primary or less educated women, in women with poorer wealth status, in contraceptive users, and in employed women. More results regarding the distribution of prevalence of pregnancy termination across the exposure and confounding variables, as well the *p*-value associated with the chi-square test, are presented in Table 2.

### 3.3. Binary Logistic Regression Models between Outcome and Exposures after Controlling for Confounders

After controlling for confounders, age group, residence, education level, wealth index, contraception use, employment status, mass media exposure (reads newspaper or magazine), and number of living children, women whose husbands exercised marital control were 26% more (adjusted odds ratio (aOR): 1.26, 95% Confidence interval (CI): 1.03–1.53) likely to experience pregnancy termination compared to those whose husbands did not exercise marital control (Table 3). Similarly, women who ever experienced sexual violence were approximately 10 times (aOR: 9.76, 95% CI: 1.91–49.96) likely to experience pregnancy termination, compared to women who did not ever experience sexual violence. Both ever experienced physical violence and emotional violence were not significantly associated with pregnancy termination in the presence of covariates.

## 4. Discussion

Termination of pregnancy is one of the most common interventions performed worldwide, Armenia inclusive. Unfortunately, studies have found that, although Armenia has a legal abortion system in place, the quality of these services remains questionable [2]. Moreover, there is compelling evidence to show that IPV exacerbates the negative effects of pregnancy termination [10,11,12]. In the context of Armenia, reviews suggest that no study has examined the relationship between intimate partner violence and pregnancy termination among Armenian women, thereby creating a literature gap. Therefore, we sought to fill this gap in the literature by examining the predictive capacity of IPV and pregnancy termination among Armenian women, using data from the 2015–2016 ADHS. 

We found that women whose husbands exercised marital control were more likely to experience pregnancy termination, compared to women whose husbands did not exercise marital control. The result is substantiated by empirical evidence that suggests that women who are not controlled in their marriages by their husbands experience greater autonomy and are able to take decisions independently, including decisions about their reproductive health, such as family planning and use of contraceptives [7,19,20,21]. As such, they are free to access and utilize family planning and contraceptives, which prevents them from even getting pregnant and, therefore, limits their potential of experiencing pregnancy termination. Another possible explanation for this finding is that the marital control exacerbates unplanned pregnancy and subsequently, pregnancy termination [22]. For example, Meiksin et al. [20] contends that marital control is associated with twice the odds of unplanned pregnancy. Therefore, in a bid to undo any inconvenience caused by an unplanned pregnancy, women in such relationships will more likely opt for pregnancy termination than women who are not controlled in their marriages. 

Furthermore, our study suggests that women who have ever experienced sexual violence are more likely to experience pregnancy termination, compared to women who did not ever experience sexual violence. Similar findings were reported by Pearson et al. [23]. The findings suggest that women who have ever experienced IPV may take conscious steps to regulate their fertility, including opting for pregnancy termination. In addition, the findings may be explained from the perspective that women who have ever experienced sexual violence are prone to pregnancy complications that cause them to opt for pregnancy termination. In some circumstances, experiences from sexual violence may even result in spontaneous abortion. This issue is supported by some studies that have shown that women who experience sexual violence can face complications that have the potential to lead to pregnancy termination [22,24]. For instance, Hall, Chappell, Parnell, Seed, and Bewley [25] found that women who experienced sexual violence, particularly rape, were more likely to experience pregnancy termination. In furtherance to the findings observed in our study, we assert that the results may be explained from the perspective that women who experience sexual violence tend to experience unintended pregnancies more than those who do not experience sexual violence [26,27,28], and most of these unintended pregnancies result in abortions [29]. Thus, potentially, sexually violated women would resort to pregnancy termination as a way of alleviating their situation. 

Again, our study suggests that although physical violence was associated with experiencing pregnancy termination, this association was not significant. This result is incongruent to the findings of studies that have found physical violence to be significantly associated with pregnancy termination. Unlike our study that found no significant association between physical violence and pregnancy termination, Ely and Murshid [30] found that there was a significant association between physical violence and pregnancy termination. Rather, the authors found no significant association between sexual violence and pregnancy termination. In contrast to our findings, Bailey [22] found that women who suffered physical violence that involved abdominal trauma while pregnant were more likely to experience ruptured uterus and membranes, which are all precursors for pregnancy termination. Again, inconsistent to our findings, several studies [19,31,32,33,34] have all found physical violence to be significantly associated with pregnancy termination. A plausible explanation for our contrasting findings may be that many Armenian women refrain from discussing the physical violence experience [35]; therefore, they do not get the necessary help that they need, including accessing health services, like pregnancy termination. Hence, the association between physical violence experience and pregnancy becomes difficult to ascertain. 

The present study demonstrates that the experience of emotional violence does not have any significant association with experiencing pregnancy termination. This finding is confirmatory of the findings of previous studies (e.g., Reference [34]) that showed that emotional violence was not significantly associated with induced abortions. However, this is inconsistent with the findings from Bola [36], who revealed that women who had ever experienced emotional violence were 33% more likely to experience pregnancy termination than women who had never experienced spousal emotional violence. This is probably because women who experience emotional violence develop a sense of emotional adequacy and incompetency to nurture a child. Therefore, they resort to pregnancy termination. 

### Strength and Limitations 

We acknowledge that the strength of our study lies in the use of a nationally representative survey that enhances the generalizability of our findings to all Armenian women. Yet, we recognize that there are some limitations in our study. The dataset used is relatively old; as such, patterns noted with current findings or outcomes may have changed over time. The use of secondary data also lacks researchers’ control. Moreover, being a cross-sectional survey, the study limits the analytical possibilities. As such, drawing causality was not possible. Further, frequency of abortion and/or miscarriage could not be presented. However, in all, we believe that our findings have practical implications for policy, program and intervention design, and implementations in Armenia.

## 5. Conclusions

Marital control and prior experience of sexual violence are associated with pregnancy termination. This draws the attention of the government and policymakers to develop and implement program that target both men and women. This will ensure that the women are empowered to take decisions about their reproductive health. Our study recognizes the influence of husbands on pregnancy termination. It signifies that policies and programs to increase awareness among husbands regarding criteria and extent of intimate partner violence should be formulated in the national health policy of Armenia and performed accordingly. In addition, influence by the relatives of husbands and women’s in-laws should be assessed and awareness should be created. Children’s cognitive development depends much on familial harmony; prevalent intimate partner violence is likely to hamper the mental growth and cognitive progress of children and adolescents. In addition, women empowerment and their decision-making power regarding reproductive health should be promoted. Therefore, it is imperative that future studies explore the extent and other forms of intimate partner violence to which husbands influence pregnancy termination decisions.

## Figures and Tables

**Table 1:** Characteristics of the respondents.

Variables	Percentage (%) (N = 2845)
Age group	
15–24	10.30
25–34	41.05
≥35	56.87
Residence	
Urban	56.87
Rural	43.12
Education level	
Primary or less	5.24
Secondary	42.14
Higher	52.62
Wealth index	
Poorest	17.79
Poorer	21.65
Middle	17.96
Richer	19.82
Richest	22.78
Reads newspaper or magazine	
Yes	47.80
No	52.20
Contraception use	
Yes	72.79
No	27.21
Employment status	
Employed	34.52
Unemployed	65.52
No. of living children	
None	6.40
One	19.02
Two	49.46
Three or more	25.13
Marital control by husband	
Yes	47.56
No	52.44
Experienced physical violence	
Yes	5.31
No	94.69
Experienced sexual violence	
Yes	0.49
No	99.51
Experienced emotional violence	
Yes	8.68
No	91.35

**Table 2:** Distribution of the prevalence of pregnancy termination across exposure and confounders.

Variables	Ever Terminated Pregnancy (%)		Chi-Square Value	*p*-Value
	Yes	No		
Age group				
15–24	16.28	83.72		
25–34	40.18	59.82	86.05	<0.01
≥35	61.41	38.59		
Residence			17.33	<0.01
Urban	44.12	55.88		
Rural	53.21	46.79		
Education level			9.45	<0.01
Primary or less	59.81	40.19		
Secondary	51.44	48.56		
Higher	44.15	55.85		
Wealth index			2.69	0.03
Poorest	51.30	48.70		
Poorer	52.27	27.73		
Middle	49.22	50.78		
Richer	43.68	46.32		
Richest	44.34	55.66		
Reads newspaper or magazine			4.01	0.046
Yes	45.73	54.27		
No	50.15	49.85		
Contraception use			59.51	<0.01
Yes	53.83	46.17		
No	32.55	67.45		
Employment status			2.12	0.15
Employed	50.59	49.41		
Unemployed	46.69	53.31		
No. of living children			74.85	<0.01
None	11.54	88.46		
One	27.82	72.18		
Two	52.07	47.93		
Three or more	64.68	35.32		
Marital control by husband			2.18	0.14
Yes	49.75	50.25		
No	46.49	53.51		
Experienced physical violence			10.56	<0.01
Yes	64.40	35.60		
No	47.12	52.88		
Experienced sexual violence			17.71	<0.01
Yes	93.44	6.56		
No	47.82	52.18		
Experienced emotional violence			8.26	<0.01
Yes	58.59	41.41		
No	47.04	52.96		

**Table 3:** Results from binary logistic regression models.

Variables	Beta Coefficients	aOR	95% CI		Wald Statistic	*p*-Value	Pseudo R^2^ (Nagelkerke)
			Lower	Upper			
**Marital control by husband**							
Yes	0.228	1.26	1.03	1.53	2.237	0.026	19.55%
No		Ref.					
**Experienced physical violence**							
Yes	0.30	1.35	0.82	2.21	1.193	0.234	19.35%
No		Ref.					
**Experienced sexual violence**							
Yes	2.28	9.76	1.91	49.96	2.736	0.007	19.56%
No		Ref.					
**Experienced emotional violence**							
Yes	0.187	1.21	0.85	1.71	1.047	0.296	19.31%
No		Ref.					

Note: Reference category for pregnancy termination is “No”. CI; Confidence interval. aOR; adjusted odds ratio.

## Data Availability

The dataset is available freely at https://dhsprogram.com/data/dataset/Armenia_Standard-DHS_2016.cfm?flag=0 (accessed on 27 March 2021).
